# A Unique Manifestation of Bardet-Biedl Syndrome with Otolaryngologic Symptoms and Bronchopneumonia in a One-year-old Girl

**DOI:** 10.7759/cureus.5717

**Published:** 2019-09-21

**Authors:** Syeda Hania Mahmood, Maria Khan, Laila Tul Qadar, Fareeha Yousuf, Mohammad Hasan

**Affiliations:** 1 Internal Medicine, Dow University of Health Sciences, Karachi, PAK; 2 Pediatrics, Dow University of Health Sciences, Karachi, PAK; 3 Internal Medicine, Jinnah Sindh Medical University, Karachi, PAK

**Keywords:** bardet-biedl syndrome, ciliopathy, hearing loss, obesity, bronchopneumonia

## Abstract

Bardet-Biedl syndrome (BBS) is a rare autosomal recessive genetic disorder. It is a congenital ciliopathy that has primary and secondary characteristics. Primary clinical features include rod-cone dystrophy, polydactyly, central obesity, genital abnormalities and mental retardation often presenting as learning difficulties. Secondary clinical features include developmental delay, speech deficit, brachydactyly/syndactyly, dental defects, ataxia, olfactory deficit, diabetes mellitus (DM) and congenital heart disease. BBS patients are friendly with a happy predisposition. Proper management, and regular examinations should be done in order to maintain healthy organ function and to avoid an early death. Renal failure is the most common cause of mortality in BBS patients.This case report illustrates the evaluation of a child with BBS, as well as the unique association of otolaryngologic symptoms and bronchopneumonia with it.

## Introduction

Bardet Biedl Syndrome (BBS) is a rare pleiotropic genetic disorder with clinical manifestations including retinal degeneration, polydactyly, obesity, hypogonadism and renal abnormalities [1]. BBS has been reported in 1:140000 to 1:160000 live births in North America and Europe, while in Kuwait and Newfoundland prevalence rate was found to be 1:13500 and 1:17500 respectively [2]. Various similarities in features exist between BBS and other syndromes. Among them, Alstrom syndrome (AS) is quite often misdiagnosed as BBS since it can also present as retinal pigmentary changes, obesity and renal impairment [3]. However, AS can be differentiated from BBS by its limited effect on cognition and absence of polydactyly [4]. Other associations that exist with BBS include Laurence-Moon syndrome (LMS), Cohen syndrome and McKusick-Kaufman syndrome. Rod cone dystrophy similar to BBS can also be present in all three syndromes, whereas polydactyly and hypogonadism are seen in LMS and McKusick-Kaufman syndrome [4]. Herein we discuss a case of BBS who presented to pediatric outpatient department (OPD) with worsening respiratory symptoms and fever.

## Case presentation

A one-year-old female infant presented to the pediatric out patient clinic of Dr. Ruth KM Pfau, Civil Hospital Karachi (CHK) with a past four-day history of difficulty in breathing, which worsened over time, and high-grade continuous fever without rigors or chills. The fever was not associated with any diarrhea, vomiting, fits or jaundice.The child was accompanied by her mother. As stated by the mother, her child had been gaining excessive weight from the age of four months while maintaining normal appetite and diet. She was exclusively breast-fed for the first four months, her mother started introducing fruit, vegetables and meat into her diet from the age of five months. A significant weight gain was observed during her first year of life, at four months she weighed 6400 g, at 11 months she weighed 14500 g (>95th percentile). The mother did not report any complications or illnesses during pregnancy. Her baby cried immediately after birth, and there were no complications during or after delivery. There is no consanguinity between mother and father. Immunization status was up to date. There was no significant family history except for her elder sibling who was obese, polydactyly of the right hand and a vision problem.

**Figure 1:**
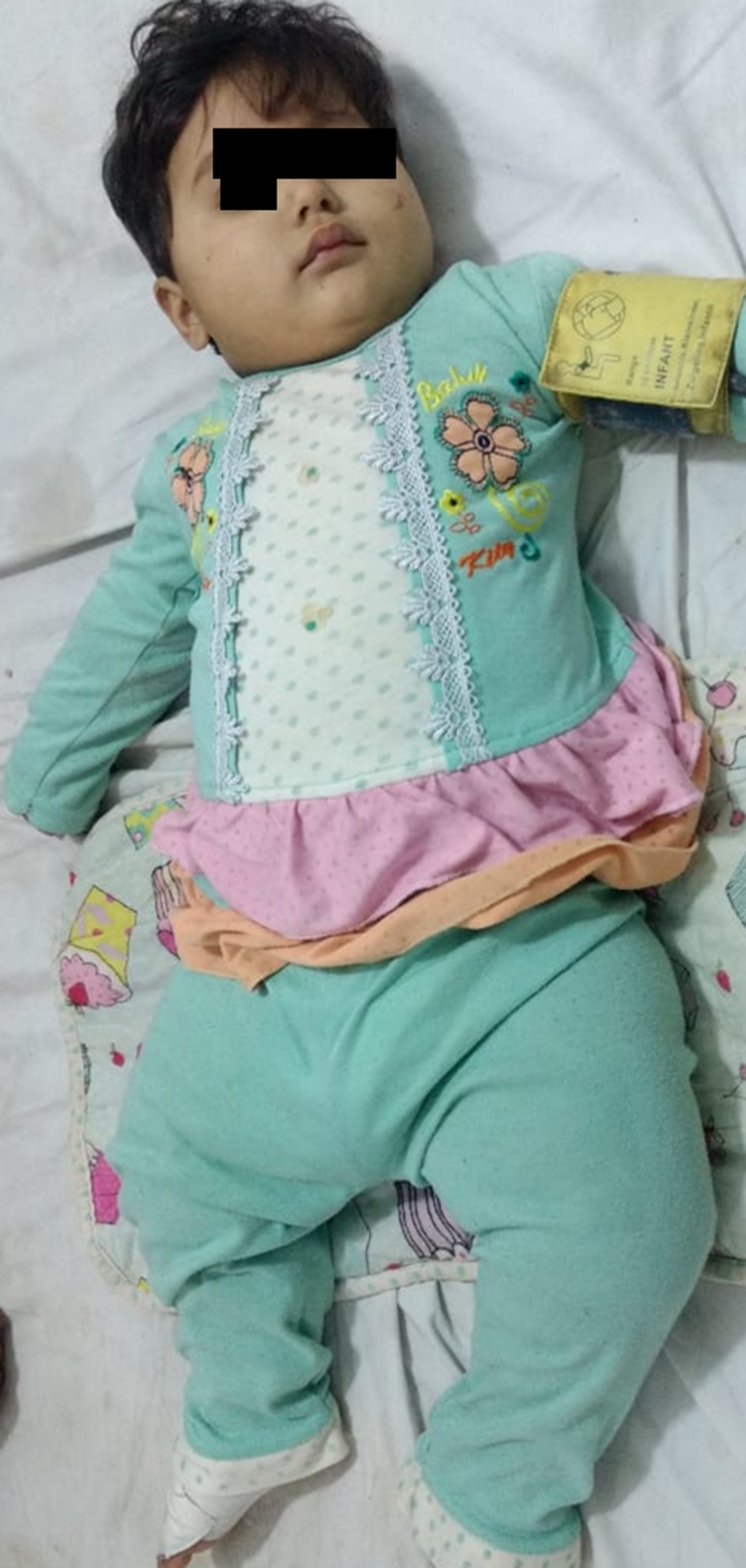
Obesity with a weight of 15000 g (>95th percentile) in our patient of BSS BSS: Bardet-Biedl Syndrome

**Figure 2:**
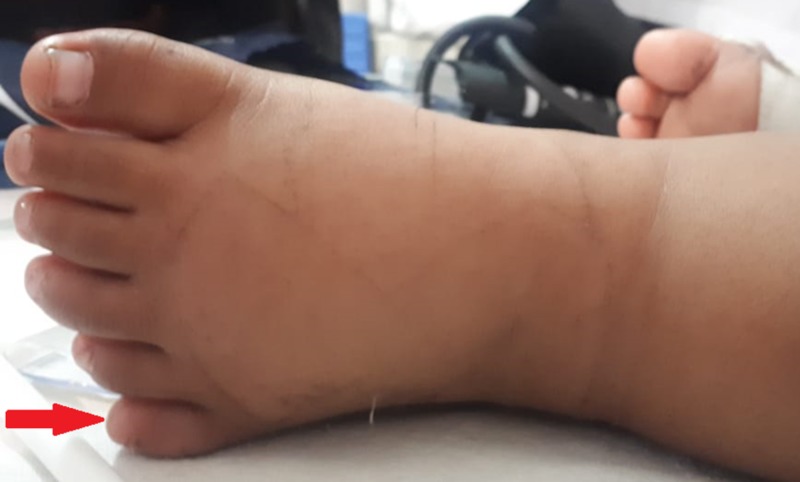
Polydactyly of left foot

On examination, the patient was lying on a bed depicting several abnormalities, including obesity with a weight of 15000 g (>95th percentile) (Figure 1), high weight for height (>98th percentile), a body mass index (BMI) was 18 (85th percentile), and also polydactyly of the left foot (Figure 2).

Her length and head circumferences were 83 cm and 46 cm respectively. She showed obvious signs of respiratory distress with a respiratory rate of 55 breaths/min and a heart rate of 112 beats/min. Her blood pressure was 127/77 mmHg. Chest examination revealed bilateral coarse crepitation with prominent subcostal and intercostal recessions. Central nervous system (CNS) examination was unremarkable with no spasticity or paraparesis. Her pupils were equally reactive to light however they were not following it. The ability of a child to follow a target with her eyes was impaired suggestive of reduced visual acuity. Rest of the systemic examination was unremarkable.

**Figure 3:**
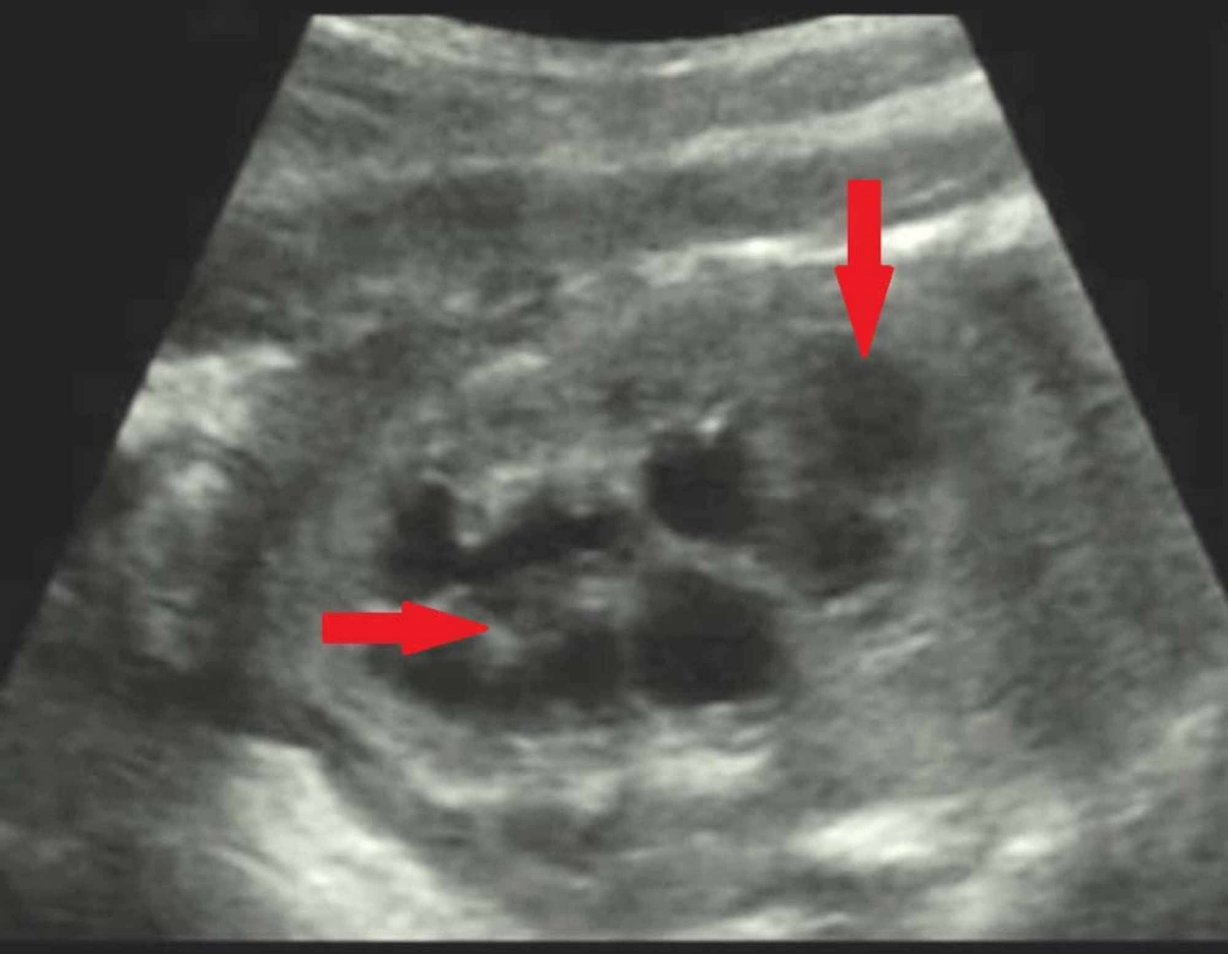
Ultrasound showing left dysplastic kidney showing multiple cysts

BBS, LMS and Cohen’s syndrome were among the differential diagnoses until further investigations were carried out. Laboratory investigation showed an elevated creatinine of 1.3 mg/dL [Normal (N) = 0.3 to 0.7], blood urea nitrogen (BUN) of 38 mg/dL (N = 4-15) and a creatinine clearance of 25.5 ml/min suggesting renal pathology. The total leukocyte count (TLC) was 14.0 x 109/L, with 40% neutrophils and 48% lymphocytes. Her inflammatory marker was also raised with a C-reactive protein (CRP) of 8.3 mg/L (N = 3) suggestive of infection. Bilateral sensory-neural hearing loss was confirmed by Brainstem Evoked Response Audiometry (BERA). The ultrasound of kidneys, ureters and bladder (KUB) showed left dysplastic kidney (Figure 3).

**Figure 4:**
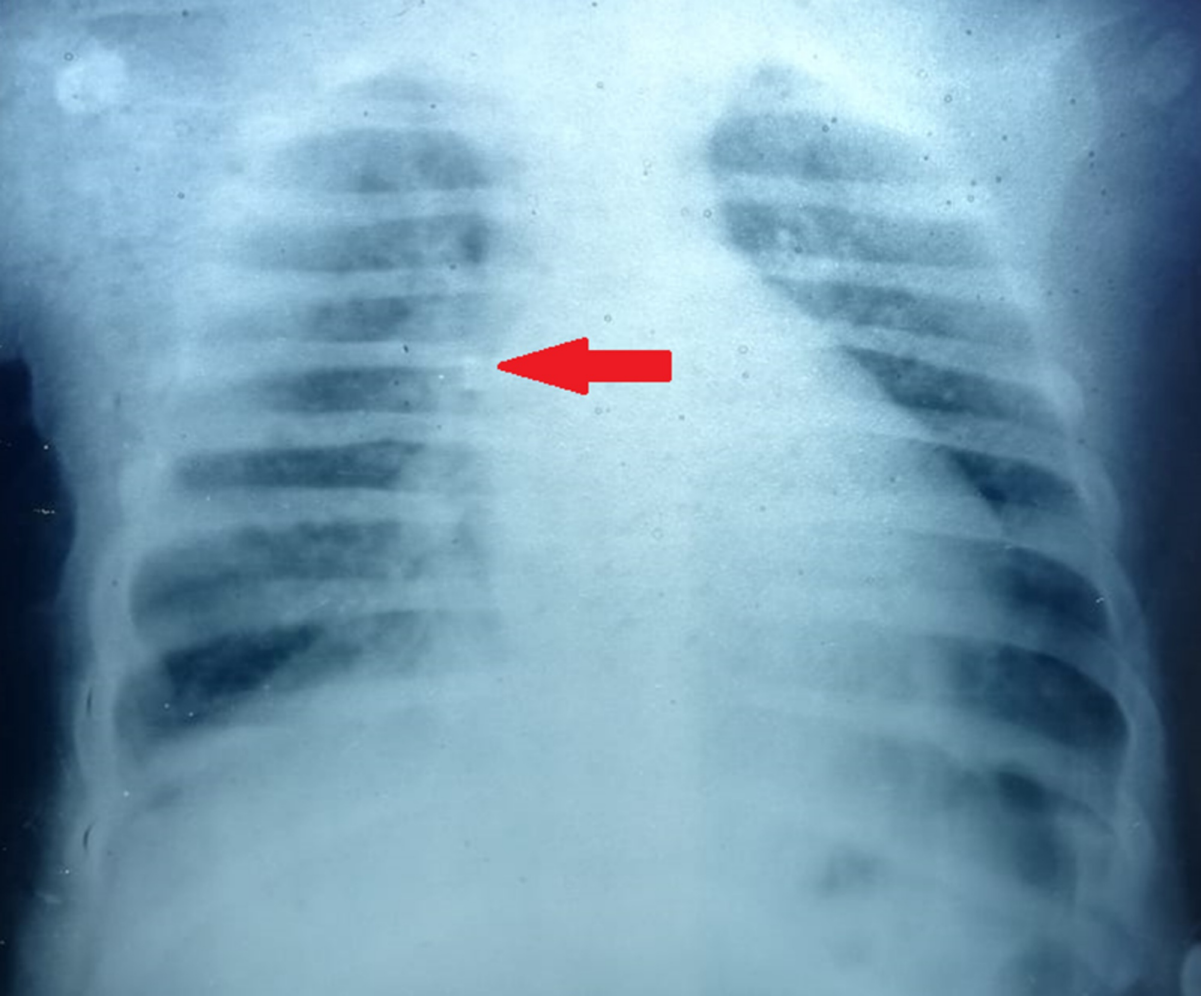
X-ray chest of our patient showing bronchopneumonia

Fundoscopy showed retinal pigmentary changes showing dark intraretinal pigments indicative of early stages of retinitis pigmentosa. The X-ray of the chest (Figure 4) confirmed the presence of bronchopneumonia. The above findings were most likely indicative of BBS in our patient.

The patient was initially treated with oxygen via continuous positive airway pressure along with intravenous (IV) amoxicillin/clavulanic acid and acetaminophen. She was nebulized with ipratropium bromide and albuterol. Her hypertension was managed by enalapril and amlodipine. She improved over a period of three days and is under regular follow up with no recurrence of respiratory complications. Interactive and play therapy was encouraged since the child was mentally challenged. She was referred to the nephrology department for the consideration of dialysis and is being evaluated by specialists to provide visual aids, hearing aids (for bilateral sensorineural hearing loss), mobility training and speech therapy. Furthermore, early intervention for the management of obesity was accomplished through proper diet and by maintaining normal appetite. The mother of a child was thoroughly counseled about these interventions. 

## Discussion

BBS is a genetic disorder with autosomal recessive inheritance. Since there are various clinical features associated with BBS, Beales et al. proposed that for diagnosis, there need to be four primary or three primary and two secondary features [5]. Primary features are characterized by rod-cone dystrophy, post-axial polydactyly, truncal obesity, learning disabilities, hypogonadism and renal anomalies. Secondary features are ataxia, behavioral abnormalities, speech delay, eye abnormalities, brachydactyly/syndactyly, mild hypertonia, diabetes mellitus, dental anomalies, cardiovascular anomalies and anosmia.

BSS is a ciliopathy in which immotile cilia or intraflagellar transport is affected [6,7]. There are diverse disorders with associated ciliopathy some of which are autosomal dominant and include recessive polycystic kidney disease, nephronophthisis and Joubert syndrome [8]. It is rare to diagnose a case of BBS in the first year of life since the age at which most cases are diagnosed is late childhood or early adulthood [9].

The first presentation of BBS is usually night blindness which is a result of pathological changes in photoreceptors [10]. Above mentioned case had retinal pigmentary changes which are found in 80-100% of cases [11]. Retinal changes are diagnosed using an electroretinogram [12]. Obesity is a cardinal feature with an incidence of 72-86% in BBS patients [9]. In most cases, children with BBS have normal birth weight. In one-third of these children, obesity appears by the age of one year [9]. Age of onset of weight gain at four months in our case is consistent with other known cases.

The most important cause of morbidity in BBS is renal failure [5]. Renal abnormalities can be dysplastic kidney, calyceal clubbing and blunting, unilateral renal agenesis, vesicoureteral reflux, scarring, hydronephrosis or horseshoe kidney [5]. According to our literature search, in 30-60% of cases, renal anomalies were a major cause of renal failure [13]. Of these anomalies mentioned, only dysplastic kidney was seen in our case. Life expectancy is significantly reduced in patients who present with renal disease.

 Treatment of BBS is mainly conservative, however, patients with polydactyly are treated surgically. For renal failure, renal replacement therapy which includes chronic peritoneal dialysis, hemodialysis and renal transplantation is the preferred choice of treatment [4]. All patients diagnosed with having BBS should be advised to have regular checkups for blood sugar levels, and renal profile. Counseling as mentioned in above case presentation is done to prevent further aggravation of the symptoms. Limitation of our case report was the unavailability of genetic testing in our healthcare institute. Reaching out overseas centers if sufficient funds are available, may provide genetic testing.

## Conclusions

The above case of BBS adds to the existing scientific literature on BBS and highlights the need to consider BBS in patients who are not a product of consanguineous marriage. Most reported cases of BBS are associated with consanguineous marriage which is an essential contributor to the incidence of BBS. The co-existence of sensorineural hearing loss is very rare; hence, it was a novel finding in our case. Since it is a genetic disorder, parents and siblings should also be inspected thoroughly for diverse symptoms of BBS. Genetic counseling of family to inform them about the possible risk of this condition in the future child is vital. Moreover, early diagnosis and proper management can improve the quality of life and prolong the lifespan.

## References

[1] Chandrasekar SP, Namboothiri S, Sen P, Sarangapani S (2018). Screening for mutation hotspots in Bardet-Biedl syndrome patients from India. Indian J Med Res.

[2] Katsanis N, Lupski JR, Beales PL (2001). Exploring the molecular basis of Bardet-Biedl syndrome. Hum Mol Genet.

[3] Dyer DS, Wilson ME, Small KW, Pai GS (1994). Alström syndrome: A case misdiagnosed as Bardet-Biedl syndrome. J Pediatr Ophthalmol Strabismus.

[4] Mihai CM, Marshall JD, Stoicescu RM (2011). Bardet-Biedl syndrome with end-stage kidney disease in a four-year-old Romanian boy: A case report. J Med Case Rep.

[5] Beales PL, Elcioglu N, Woolf AS, Parker D, Flinter FA (1999). New criteria for improved diagnosis of Bardet-Biedl Syndrome: results of a population survey. J Med Genet.

[6] Tobin JL, Beales PL (2007). Bardet-Biedl syndrome: beyond the cilium. Pediatr Nephrol.

[7] Panny A, Glurich I, Haws RM, Acharya A (2017). Oral and craniofacial anomalies of Bardet-Biedl syndrome: dental management in the context of a rare disease. J Dent Res.

[8] Kagan KO, Dufke A, Gembruch U (2017). Renal cystic disease and associated ciliopathies. Curr Opin Obstet Gynecol.

[9] Green JS, Parfrey PS, Harnett JD (1989). The cardinal manifestations of Bardet-Biedl syndrome, a form of Laurence-Moon-Biedl syndrome. N Engl J Med.

[10] Wheway G, Schmidts M, Mans DA (2015). An siRNA-based functional genomics screen for the identification of regulators of ciliogenesis and ciliopathy genes. Nat Cell Biol.

[11] Garg M, Madhu S, Dwarakanath C, Ammini A (1998). Laurence-Moon-Bardet-Biedl syndrome - presenting with acute onset of diabetes mellitus. Med J Armed Forces India.

[12] Baker K, Beales PL (2009). Making sense of cilia in disease: the human ciliopathies. Am J Med Genet Part C Semin Med Genet.

[13] Sowjanya B, Sreenivasulu U, Naidu JN, Sivaranjani N (2011). End stage renal disease, differential diagnosis, a rare genetic disorder: Bardet-biedl syndrome: Case report and review. Indian J Clin Biochem.

